# Trend and burden of adult cancer-related hospitalizations in the United States

**DOI:** 10.1038/s41598-025-97310-x

**Published:** 2025-04-18

**Authors:** Muni Rubens, Sandeep Appunni, Anshul Saxena, Venkataraghavan Ramamoorthy, Atulya Aman Khosla, Peter McGranaghan, Mayur Doke, Veda Rabishanker, Yanjia Zhang

**Affiliations:** 1 Office of Clinical Research, Miami Cancer Institute, Baptist Health South Florida, Office of Clinical Research, Miami, FL 33176 USA; 2 Herbert Wertheim College of Medicine, Florida International University, Miami, FL 33176 USA; 3 Universidad Espiritu Santo, Samborondón, Ecuador; 4 Government Medical College, Kozhikode, Kerala India; 5 Baptist Health South Florida, Miami, FL USA; 6 William Beaumont University Hospital, Royal Oak, MI 48073 USA; 7 Semmelweis Doctoral College, Semmelweis University, Budapest, Hungary; 8 University of Miami, Miami, FL USA; 9 McNeil HS, Austin, TX USA; 10 Department of Internal Medicine and Cardiology, Charité-Universitätsmedizin Berlin, Corporate Member of Freie Universität Berlin and Humboldt Universität zu Berlin, Augustenburger Platz 1, 10117 Berlin, Germany

**Keywords:** Cancer, Trends, Hospitalization, Cost, Mortality, Disposition, Length of stay, Cancer, Oncology

## Abstract

To calculate national trends and estimates of cancer-related hospitalizations, hospital outcomes, and hospitalization costs. The current study was a retrospective analysis of National Inpatient Sample data, collected during 2008 to 2019. Adult cancer hospitalizations were identified by Clinical Classifications Software codes. We estimated weighted frequencies, proportions, and trends in cancer-related hospitalizations, hospital outcomes, and hospitalization costs. The primary outcome of the study was cancer-related hospitalizations, and secondary outcomes were the reasons for hospitalization, disposition status, hospital length of stay, and hospitalization cost. There were 371 million weighted hospitalizations during 2008 to 2019, of which 15.1% (56 million) were cancer related. The most common cancer types were breast cancer (11.9%), secondary malignancies (11.2%), and prostate cancer (10.3%), and the most common reasons for cancer-related hospitalizations were septicemia (4.8%), pneumonia (4.7%), and complications of surgical procedures or medical care (3.1%). Trend analysis showed that the total number of cancer-related hospitalizations increased from 12,963 to 16,500 per 100,000 hospitalizations during the study period (relative increase, 27.3%). Mortality rates decreased from 5.1% to 4.0% (relative decrease, 21.6%), while hospital length of stay decreased from 3.5 to 3.2 days (relative decrease, 8.6%), during the study period. Disposition to home or short-term facilities decreased (relative decrease, 3.1%), while to long-term facilities increased (relative increase, 20.6%) during the same period. Total hospitalization cost increased from $55.5 billion in 2008 to $76.4 billion in 2019 (relative increase, 37.7%). Our study provides preliminary estimates and findings for developing an evidence base for understanding cancer-related hospitalizations.

**Supplementary Information:**

The online version contains supplementary material available at 10.1038/s41598-025-97310-x.

## Introduction

Cancer has consistently been one of the leading causes of morbidity and mortality in the US. In 2024 alone, there were an estimated 2 million new cancer cases and nearly 611,720 cancer deaths^1^. Although the incidence of cancer has been declining over the past couple of decades, the number of cancer survivors continue to increase^2^. There were an estimated 16.9 million cancer survivors in 2019, and this number is projected to increase to 22.2 million by the year 2030^1^. This is primarily due to early detection, better treatment, and an aging population.

Cancer is associated with substantial financial cost and healthcare expenditure. In 2015, about $183 billion was spent on cancer-related healthcare, and this amount is projected to increase to $246 billion by 2030^3^. Hospitalization constitutes one of the primary drivers of cancer-related healthcare expenditure^4,5^ and typically occur primarily during the initial year after diagnosis and the final year before death^6^. Accumulating evidence shows that cancer-related hospitalization expenditure could be substantially decreased through improved outpatient services, and early and judicious palliative care referral^7,8^. Therefore, decreasing unnecessary cancer-related hospitalizations has been increasingly viewed as a possible solution for decreasing cost and improving quality of cancer care.

Decreasing the number of unnecessary cancer hospitalizations is also an important strategy for improving the access to and availability of high-quality, patient-centered cancer care. Understanding the burden and characteristics of cancer-related hospitalizations could be helpful to appropriately direct resources, improve outcomes, and avoid unnecessary hospitalizations. An initial step in this direction would be to identify the characteristics and clinical presentations of cancer patients admitted to the hospitals as well as review their in-patient outcomes.

However, there are currently only few studies that have examined these characteristics among cancer hospitalizations. Existing studies are small-scale and have focused on hospitalization for specific types of cancers in selected institutions^9–14^. Therefore, the objective of this study was to evaluate the national trends, estimates, and reasons for cancer-related hospitalizations using a nationally representative database. In addition, we also analyzed additional variables such as disposition status, hospital length of stay, and associated hospitalization cost, to identify opportunities for improvements.

## Methods

### Study design and data source

The current study was a retrospective analysis of National Inpatient Sample (NIS) data, collected between 2008 and 2019. NIS is the largest all-payer in-patient database developed by a Federal-State-Industry partnership by the Agency of Healthcare Research and Quality (AHRQ)^15^. The NIS data is collected from hospitals in the states participating in the Healthcare Cost and Utilization Project (HCUP), an initiative by the AHRQ. NIS collects and stores data from more than 35 million weighted hospitalizations within the US annually. This includes collecting a 20% stratified sample of all discharge data from all US community hospitals, except long-term acute care hospitals and rehabilitation centers. Each hospitalization recorded in the NIS comprises of one primary and up to 40 secondary diagnoses. Other available data include demographics, insurance data, clinical procedures, comorbidities, hospital length of stay, disposition status, and hospitalization cost. The primary outcome of the study was cancer-related hospitalizations. The secondary outcomes were reason for hospitalization, disposition status, hospital length of stay, and hospitalization cost.

### Study variables

All cancer-related hospitalizations were identified using validated Clinical Classifications Software (CCS) codes (primary and secondary diagnosis). The NIS dataset records up to 40 CCS diagnosis codes for each hospitalization, and cancer-related hospitalizations were identified by CCS codes 11 to 45, as described previously^16^. All patients ≥ 18 years of age were included in the analyses. Among these patients, the first listed non-cancer CCS code was used for identifying the primary reason for hospitalization. The CCS codes were developed as an extension of the International Classification of Diseases, Clinical Modification codes with an intent to develop a smaller subset of clinical categories that may be useful for meaningful interpretation. The CCS diagnosis code was selected as the preferred variable to identify cancer-related hospitalization due to its clinical application and analytical feasibility. We categorized the discharge disposition information in the NIS into three groups: (1) home or short-term facility; (2) long-term facility; and (3) death (died in the hospital).

### Statistical analysis

The guidelines for using NIS data developed by Khera and Krumholz were used to ensure appropriate procedures for the study^17^. The NIS was redesigned in 2012 to improve national estimates. To account for this redesigning, we used modified discharge weights for the years 2008–2011^18^. Initially, we compared cancer-related versus non-cancer-related hospitalizations by demographics and hospital characteristics. We classified cancer-related hospitalizations by cancer type and calculated the proportion of hospitalization for each cancer type. Subsequently, we calculated disposition status, hospital length of stay, and hospitalization cost for each cancer type. Similarly, among cancer-related hospitalization, we identified the top 10 primary reasons for hospitalization based on weighted frequencies. We then calculated disposition status, hospital length of stay, and hospitalization cost for each of the top 10 primary reasons for hospitalization. We calculated trends in cancer-related hospitalization, hospital length of stay, in-hospital mortality, and hospitalization cost, during 2008–2019. The hospitalization cost for each year was adjusted according to 2019 inflation levels released by the US Consumer Price Index^19^. Cancer hospitalization trend was assessed using linear regression. For each estimate, we calculated the relative standard error and considered values greater than 30% to be of low precision. Sample weights were used for all analyses to accurately represent national estimates for the evaluated parameters. All results were reported as weighted estimates. Statistical analysis was performed using SAS (SAS Institute, Cary, NC).

## Results

### Demographics and hospital characteristics

A total of 371,446,842 weighted hospitalizations were reported between 2008 and 2019, of which 15.1% (56,143,160) were cancer-related hospitalizations. Patients with cancer-related hospitalizations were older, male, white, and had higher proportion of Medicare insurance, compared to non-cancer-related hospitalizations. The distribution of other demographic and hospital characteristics did not differ considerably between the two groups (Table 1).

**Table 1:** Comparison of cancer-related versus non-cancer-related hospitalizations in the United States, 2008 to 2019.

Characteristics	Cancer-related hospitalizations (n = 56,143,160, 15.1%)	Non-cancer-related hospitalizations (n = 315,303,682, 84.9%)	P value
	Weighted frequency (%)		
Age			< 0.001
18–44 years	4,030,779 (7.2)	106,921,508 (33.9)	
45–64 years	16,845,767 (30.0)	90,091,324 (28.5)	
65–74 years	14,091,981 (25.1)	46,579,834 (14.7)	
≥ 75 years	21,174,633 (37.7)	71,711,016 (22.7)	
Sex			< 0.001
Male	27,260,979 (48.5)	122,422,828 (38.8)	
Female	28,853,156 (51.3)	192,582,249 (61.0)	
Missing	29,025 (0.1)	298,606 (0.1)	
Race			< 0.001
White	38,156,867 (68.0)	182,651,583 (57.9)	
Black	5,441,716 (9.7)	40,680,373 (12.9)	
Hispanic	3,338,647 (5.9)	31,155,350 (9.9)	
Asian or Pacific Islander	1,083,864 (1.9)	6,680,024 (2.1)	
Native American	207,906 (0.4)	1,875,834 (0.6)	
Other	1,155,120 (2.1)	8,502,486 (2.7)	
Missing	6,759,040 (12.0)	43,758,032 (13.9)	
Insurance			< 0.001
Medicare	35,030,245 (62.3)	133,759,724 (42.4)	
Medicaid	4,257,279 (7.5)	54,212,921 (17.1)	
Private	14,231,128 (25.3)	96,586,104 (30.6)	
Uninsured	1,301,974 (2.3)	19,317,410 (6.1)	
Other	1,239,568 (2.2)	10,800,639 (3.4)	
Missing	82,964 (0.1)	626,886 (0.1)	
Income			< 0.001
Quart 1	13,987,600 (24.9)	92,730,254 (29.4)	
Quart 2	13,870,904 (24.7)	80,110,643 (25.4)	
Quart 3	13,657,700 (24.3)	72,730,310 (23.0)	
Quart 4	13,456,435 (23.9)	62,046,942 (19.6)	
Missing	1,170,520 (2.0)	7,685,532 (2.4)	
Admission type			< 0.001
Elective	40,157,335 (71.5)	233,533,537 (74.0)	
Non-elective	15,824,123 (28.1)	80,746,672 (25.6)	
Missing	161,702 (0.2)	1,023,474 (0.3)	
Hospital bed size			< 0.001
Small	7,330,844 (13.0)	44,672,856 (14.1)	
Medium	13,565,797 (24.1)	82,538,684 (26.1)	
Large	35,042,658 (62.4)	186,824,225 (59.2)	
Missing	203,861 (0.3)	1,267,917 (0.4)	
Hospital region			< 0.001
Northeast	11,798,517 (21.0)	60,681,111 (19.2)	
Midwest	13,412,629 (23.8)	71,912,246 (22.8)	
South	20,484,070 (36.4)	122,937,391 (38.9)	
West	10,447,943 (18.6)	59,772,934 (18.9)	
Location and teaching status			< 0.001
Rural	5,541,401 (9.8)	37,988,628 (12.0)	
Urban non-teaching	19,595,362 (34.9)	121,739,880 (38.6)	
Urban teaching	30,802,536 (54.8)	154,307,257 (48.9)	
Missing	203,861 (0.3)	1,267,917 (0.4)	
Disposition			< 0.001
Home or short-term facility	43,361,957 (77.3)	262,137,730 (83.2)	
Long-term facility	10,303,705 (18.4)	47,177,885 (15.0)	
Death	2,446,899 (4.3)	5,808,441 (1.8)	
Death			< 0.001
Yes	2,446,899 (4.3)	5,808,441 (1.8)	
No	53,665,431 (95.6)	309,312,106 (98.1)	
Missing	30,829 (0.1)	183,135 (0.1)	
Length of stay in days, median (IQR)	3.3 (1.7–6.2)	2.5 (1.3–4.7)	< 0.001
Hospitalization cost in USD, median (IQR)	9846 (5600–17,598)	12,982 (9282–14,271)	< 0.001

### Cancer-related hospitalizations

The most common cancer types were breast cancer (11.9%), secondary malignancies (11.2%), and prostate cancer (10.3%). Cancer types associated with the greatest mortality were liver cancer (9.4%), secondary malignancies (8.5%), and pancreatic cancer (8.4%). Cancer types with the highest median hospital length of stay were multiple myeloma (4.5 days), gastrointestinal cancer (4.4 days), pancreatic cancer (4.4 days), and rectal cancer (4.4 days). Cancer types with the greatest median hospitalization cost were cancer of brain and nervous system ($13,686), rectal cancer ($11,878), and cancer of the bone and connective tissue ($11,769). Cancer-related hospitalization by cancer type and associated disposition status, hospital length of stay, and hospitalization cost are reported in Table 2.

**Table 2:** Cancer-related hospitalization by cancer type and associated disposition status, length of stay, and hospitalization cost.

Cancer type	Weighted frequency (%)	Disposition status, %			Length of stay in days, median (IQR)	Hospitalization cost in USD, mean (IQR)
		Home or short-term facility	Long-term facility	Death		
Breast	6,667,921 (11.9)	76.5 (76.3–76.8)	21.5 (21.2–21.8)	2.0 (1.9–2.0)	2.6 (1.3–4.7)	8602 (5154–14,746)
Secondary malignancies	6,293,580 (11.2)	72.2 (71.7–72.7)	19.3 (18.8–19.7)	8.5 (8.4–8.6)	4.1 (2.1–7.5)	10,678 (6048–19,310)
Prostate (and other male genital cancers)	5,799,555 (10.3)	78.6 (78.3–78.9)	19.2 (18.9–19.4)	2.2 (2.2–2.3)	2.5 (1.2–4.7)	9423 (5494–15,732)
Lung (and other respiratory cancers)	5,175,152 (9.2)	73.8 (73.6–74.1)	18.0 (17.8–18.2)	8.2 (8.1–8.3)	3.9 (2.0–6.9)	10,076 (5631–18,138)
Colon	4,195,345 (7.5)	74.6 (74.4–74.9)	21.9 (21.7–22.2)	3.4 (3.4–3.5)	3.9 (2.1–6.8)	10,310 (5699–18,171)
Cancer of unknown or unspecified origin	3,837,420 (6.8)	75.9 (75.7–76.1)	20.3 (20.1–20.5)	3.8 (3.7–3.9)	3.6 (1.8–6.7)	9638 (5524–17,540)
Female reproductive	3,235,083 (5.8)	84.1 (83.8–84.3)	13.9 (13.7–14.2)	2.0 (2.0–2.1)	2.9 (1.5–5.1)	9040 (5324–15,254)
Melanoma (and other skin cancers)	2,794,122 (5.0)	76.6 (76.3–76.8)	21.6 (21.3–21.8)	1.9 (1.8–1.9)	2.7 (1.4–4.8)	9101 (5269–15,866)
Non-Hodgkin lymphoma	2,343,342 (4.2)	76.9 (76.6–77.2)	17.7 (17.5–18.0)	5.4 (5.3–5.4)	3.8 (2.0–7.4)	10,476 (5760–20,169)
Leukemia(s)	2,090,661 (3.7)	75.5 (75.1–75.9)	17.0 (16.6–17.4)	7.5 (7.4–7.6)	4.1 (2.0–8.3)	10,729 (5755–22,359)
Bladder (and other urinary organ cancers)	1,849,639 (3.3)	76.7 (76.4–77.0)	20.5 (20.2–20.7)	2.8 (2.8–2.9)	3.3 (1.6–6.1)	9107 (5267–16,979)
Gastrointestinal	1,674,900 (3.0)	77.7 (77.3–78.0)	16.4 (16.1–16.7)	5.9 (5.8–6.0)	4.4 (2.2–8.1)	11,171 (6020–21,680)
Kidney and renal	1,554,847 (2.8)	83.0 (82.8–83.3)	14.5 (14.3–14.8)	2.4 (2.4–2.5)	3.1 (1.7–5.3)	10,753 (6219–17,404)
Active chemotherapy treatment (not associated with a specific cancer site)	1,502,163 (2.7)	95.9 (95.6–96.1)	3.2 (3.0–3.4)	1.0 (0.9–1.0)	3.4 (1.9–4.8)	9965 (5932–16,967)
Head and neck	1,234,065 (2.2)	79.5 (79.1–79.8)	17.0 (16.7–17.4)	3.5 (3.4–3.6)	3.5 (1.7–6.8)	10,128 (5600–19,348)
Rectal	1,205,175 (2.1)	82.1 (81.7–82.4)	15.2 (14.9–15.5)	2.7 (2.6–2.8)	4.4 (2.4–7.5)	11,878 (6449–20,774)
Multiple myeloma	1,048,431 (1.9)	73.7 (73.2–74.2)	20.4 (19.9–20.9)	5.9 (5.7–6.0)	4.5 (2.2–8.7)	11,006 (5983–21,961)
Pancreatic	872,057 (1.6)	75.9 (75.5–76.4)	15.7 (15.3–16.0)	8.4 (8.2–8.6)	4.4 (2.2–7.9)	10,717 (5814–20,389)
Brain and nervous system	728,514 (1.3)	70.4 (69.9–71.0)	25.7 (25.2–26.1)	3.9 (3.8–4.1)	3.8 (1.9–7.4)	13,686 (6634–25,649)
Thyroid	720,287 (1.3)	90.1 (89.8–90.4)	8.9 (8.6–9.2)	1.0 (0.9–1.0)	1.8 (0.8–3.5)	7924 (4845–13,265)
Liver	713,276 (1.3)	76.4 (75.9–76.9)	14.2 (13.8–14.6)	9.4 (9.2–9.7)	3.7 (1.8–6.8)	10,259 (5659–19,244)
Hodgkin’s disease	325,853 (0.6)	86.1 (85.8–86.5)	9.9 (9.6–10.3)	3.9 (3.8–4.1)	3.3 (1.6–6.7)	9921 (5385–20,053)
Cancer of bone and connective tissue	281,773 (0.5)	81.5 (80.7–82.2)	14.9 (14.3–15.6)	3.6 (3.4–3.8)	3.9 (1.9–7.2)	11,769 (6547–22,849)

The most common reasons for cancer-related hospitalization were septicemia (4.8%), pneumonia (4.7%), and complications of surgical procedures or medical care (3.1%). Among the top ten reasons for cancer-related hospitalization, septicemia had the highest mortality rate (18.0%), greatest median hospital length of stay (5.2 days), and highest median hospitalization cost ($12,727). Table 3 shows the top 10 reasons for cancer-related hospitalization and associated disposition status, hospital length of stay, and hospitalization cost.

**Table 3:** Top 10 reasons for cancer-related hospitalization, and associated disposition status, length of stay and hospitalization cost.

Cancer type	Weighted frequency (%)	Disposition status, %			Length of stay in days, median (IQR)	Hospitalization cost in USD, median (IQR)
		Home or short-term facility	Long-term facility	Death		
Septicemia	2,688,575 (4.8)	54.7 (54.4–55.0)	27.3 (27.1–27.6)	18.0 (17.8–18.2)	5.2 (2.8–9.3)	12,727 (7233–24,073)
Pneumonia	2,626,718 (4.7)	72.0 (71.8–72.3)	21.0 (20.7–21.3)	7.0 (6.9–7.1)	4.3 (2.5–7.4)	9220 (5570–16,377)
Complications of surgical procedures or medical care	1,729,051 (3.1)	84.8 (84.5–85.1)	13.4 (13.1–13.7)	1.8 (1.8–1.9)	4.2 (2.1–7.5)	11,470 (6140–21,184)
Congestive heart failure	1,664,021 (3.0)	74.5 (74.2–74.8)	21.4 (21.1–21.6)	4.1 (4.1–4.2)	3.6 (2.0–6.1)	8195 (5126–14,013)
Chronic obstructive pulmonary disease and bronchiectasis	1,596,275 (2.9)	83.6 (83.3–83.8)	13.8 (13.5–14.0)	2.7 (2.6–2.8)	3.3 (1.9–5.5)	7675 (4811–12,901)
Fluid and electrolyte disorders	1,471,349 (2.7)	77.1 (76.7–77.4)	18.4 (18.0–18.7)	4.6 (4.5–4.7)	3.1 (1.7–5.7)	7092 (4070–13,417)
Acute and unspecified renal failure	1,466,439 (2.6)	68.0 (67.7–68.3)	24.3 (24.0–24.6)	7.7 (7.6–7.9)	4.5 (2.4–8.0)	9689 (5415–18,553)
Cardiac dysrhythmias	1,465,863 (2.6)	85.2 (85.0–85.4)	12.7 (12.5–12.9)	2.1 (2.0–2.1)	2.6 (1.2–4.7)	8212 (4634–15,635)
Intestinal obstruction without hernia	1,360,257 (2.5)	84.8 (84.6–85.1)	12.3 (12.1–12.5)	2.9 (2.8–2.9)	4.3 (2.3–7.9)	9312 (5077–18,518)
Deficiency and other anemia	1,256,567 (2.3)	84.6 (84.3–84.9)	12.7 (12.4–13.0)	2.6 (2.6–2.7)	3.3 (1.5–6.6)	8799 (4767–17,543)

Septicemia (except in labor).

Pneumonia (except that caused by tuberculosis or sexually transmitted disease).

Chronic obstructive pulmonary disease and bronchiectasis.

Congestive heart failure; non-hypertensive.

The primary reason for cancer-related hospitalization by cancer type are shown in Supplementary Table 1. The top 2 primary reasons for hospitalization among breast cancer were osteoarthritis (5.0%) and congestive heart failure (3.9%); among secondary malignancies were septicemia (6.7%) and other nervous system disorders (5.8%). Among prostate cancer, the top 2 reasons for hospitalization were osteoarthritis (4.2%) and congestive heart failure (3.9%); among lung cancer, pneumonia (12.8%) and chronic obstructive pulmonary disease (9.3%); and among colon cancer, intestinal obstruction without hernia (7.5%) and complications of surgical procedures or medical care (4.1%). Among hospitalization where chemotherapy or radiotherapy was reported, the primary reason for hospitalization was deficiency and other anemia (10.3%), essential hypertension (7.7%), and fluid and electrolyte disorders (3.6%) (Supplementary Table 2).

### Trends in cancer-related hospitalizations and hospital outcomes

**Fig. 1:**
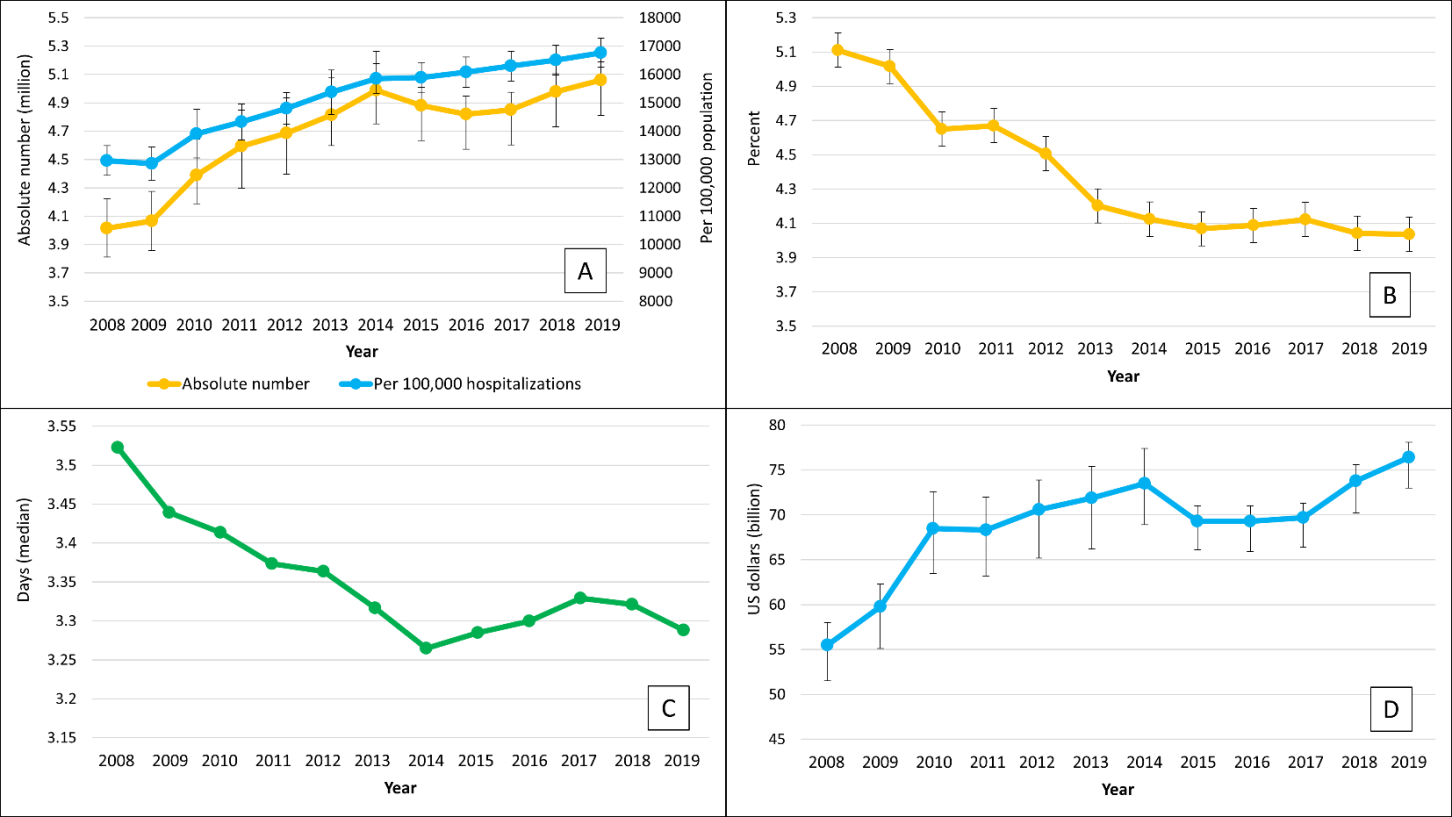
Trends in cancer-related hospitalization rate (**A**), in-hospital mortality rate (**B**), hospital length of stay (**C**), and total hospitalization cost (**D**).

The total number of cancer-related hospitalizations increased from 4.0 million in 2008 to 5.0 million in 2019. This translated to an increase from 12,963 to 16,500 per 100,000 hospitalizations (relative increase, 27.3%) (Fig. 1A). Among different cancer types, the greatest increasing trend was observed for melanoma (relative increase, 119.6%), liver cancer (relative increase, 71.1%), and cancer of unknown or unspecified origin (relative increase, 36.7%), while the greatest decreasing trend was observed for maintenance chemotherapy and radiotherapy (relative decrease, 26.7%), colon cancer (relative decrease, 23.8%), and lung cancer (relative decrease, 23.4%). Trends of individual cancer types during the study period are shown in Supplementary Table 3.

Trends in disposition status showed that disposition to long-term facilities increased from 16.5% to 19.9% during the study period (relative increase, 20.6%). Disposition to home or short-term facility decreased from 78.4% to 76.0% (relative decrease, 3.1%) and disposition classified as death decreased from 5.1% to 4.0% (relative decrease, 21.6%), during the same period (Fig. 1B).

Hospital length of stay showed decreasing trends from 3.5 to 3.2 days (relative decrease, 8.6%) during the study period (Fig. 1C). Total hospitalization cost increased from $55.5 billion in 2008 to $76.4 billion in 2019 (relative increase, 37.7%). (Fig. 1D).

## Discussion

To the best of our knowledge, this is the first study to report national trends in cancer-related hospitalizations using a nationally representative database such as the NIS. Our study found that over 4.7 million cancer-related hospitalization occur every year in the US. There were 4 million cancer-related hospitalization in 2008, which had increased to more than 5 million by 2019, resulting in an increase in hospitalization cost from 55.5 to 76.4 billion during the study period. Patients with cancer-related hospitalization were more likely to be older, male, white, and have Medicare coverage, compared to patients with non-cancer-related hospitalization. In addition, they also had greater hospital length of stay, mortality, adverse disposition status, and higher hospitalization cost. Cancer treatment involves not only treating the cancer itself but also managing treatment-related complications and associated comorbidities which requires a multidisciplinary approach. In addition, the presence of cancer or even a history of cancer could complicate hospital outcomes of patients hospitalized for reasons other than cancer itself. Understanding these complexities and population characteristics are important for improving care delivery and patient outcomes.

In our study, common cancer types among cancer-related hospitalization were breast, prostate, and lung cancer, mirroring the prevalence of these cancer types in the US population^2,20^. The higher volume of hospitalization related to secondary malignancies reflects the increasing number of cancer survivors and greater prevalence of cancer due to the aging population in the US.

We found that the number of cancer-related hospitalizations, disposition to long-term facilities, and hospitalization costs increased during the study period. An increased hospital burden and care cost could adversely affect access and availability of high-quality cancer care. However, increasing trends in disposition status to long-term facilities also imply that a greater number of elderly cancer patients are surviving advanced stages of the disease and receiving better levels of care. We also found that mortality and hospital length of stay showed decreasing trends during the study period. Hospitalization cost increased despite decreasing trends in the hospital length of stay, probably due to increased rates of treatment deliveries such as chemotherapies, radiotherapies, and surgeries, and higher level of healthcare system utilization, which could have translated to decreasing mortality rates. Other reasons for decreased mortality could be earlier diagnosis due to improved access to the healthcare system, leading to better screening initiatives and early symptom evaluation. The increase in hospitalization cost could also be due to the increasing number of hospitalizations, as observed in our study. Although the incidence of cancer has been decreasing over the last two decades, with the aging population, better screening and diagnosis, and improved cancer management strategies, the current increasing trends in cancer-related hospitalization and hospital outcomes are expected to continue.

Infectious causes such as septicemia and pneumonia were among the top five primary reasons for cancer-related hospitalization. Respiratory causes such as chronic obstructive pulmonary disease and bronchiectasis, and gastrointestinal causes such as intestinal obstruction were among the top ten primary reasons for hospitalization. In a large-scale study done by Rivera et al., infectious, respiratory, and gastrointestinal causes were among the most common reasons for cancer-related emergency department visits and subsequent hospitalizations^16^. Similarly, in another study that looked for emergency department visits, respiratory and gastrointestinal causes were predominant among cancer-related visits^21^. Cancer and its associated chemotherapy, radiotherapy, undernutrition, and comorbidities significantly impair immunity and precipitate infections^22^. The respiratory and gastrointestinal causes could also be due to tumors in these sites or secondary to treatment complications. The predominance of respiratory and gastrointestinal causes among cancer-related hospitalization reflect the greater prevalence of lung and colon cancers in the US population^2^.

We found that age-related chronic diseases such as CHF, cardiac dysrhythmias, and COPD were among the top ten reasons for cancer-related hospitalization. Cancer largely being a disease of the elderly, co-existing chronic diseases are common in this population. Both cancer and its treatment could exacerbate these chronic diseases and potentially complicate the clinical condition, resulting in hospitalization. Comorbidities are independent predictors of mortality among cancer patients, especially among the elderly^23^.

We used CCS codes for identifying cancer-related hospitalization, which capture different cancer types and patients in all stages of the disease continuum. However, the CCS code for maintenance chemotherapy or radiotherapy does not explicitly indicate a cancer type or treatment-related event. When we looked for the primary reason for hospitalization among patients with maintenance chemotherapy or radiotherapy, anemia, electrolyte imbalance, coagulation disorders, nutritional disorders, and metabolic disorders were the common reasons for hospitalization. These are all common adverse events of cancer treatment^24^. CCS codes for maintenance chemotherapy or radiotherapy were commonly associated with hospitalization for lung cancer and colon cancer.

We found that 4.3% of cancer-related hospitalization terminated in mortality, which was 2.4 times greater than the rates among non-cancer-related hospitalization. Similarly, hospital length of stay, disposition to long-term facilities, and hospitalization cost were greater for cancer-related hospitalization. Mortality rates were highest for patients with secondary malignancies, liver cancer, and pancreatic cancer. Secondary malignancies indicate advanced stages of the disease, and liver and pancreatic cancers have lower overall survival rates.

In our study, multiple myeloma, gastrointestinal cancer, pancreatic cancer, and rectal cancer had the longest hospital length of stay. These cancer types require prolonged hospitalization for managing acute illness, adverse events of outpatient treatments, induction chemotherapy, stem cell transplantation, and complications of surgical and medical treatment. Higher mortality rates, greater rates of transfers to long-term facilities, greater hospital length of stay, and higher hospitalization cost in our study indicate that these patients were sicker, frail, and had advanced disease.

Decreasing hospitalization is important for cancer care because it will reduce health care cost, improve the access and quality of treatment to the neediest, and improve the quality of life for cancer patients. Although there are initiatives to decrease preventable cancer hospitalization, there are currently no validated administrative measures or clinical matrices. This is challenging because cancer is a complex and multifaceted construct. In addition, interventions to decrease avoidable cancer hospitalization should not affect the therapeutic relationship between patient and oncologist and patient-centered cancer care. Our study provides preliminary estimates and findings for developing an evidence base for decreasing cancer-related hospitalization and assessing the effectiveness of initiatives to decrease them in the future. Based on our findings, treatment complications, infections, comorbidities, and cardiac and pulmonary diseases should be targeted for future preventive and interventional strategies to reduce hospital burden. Oncologists and other specialists could incorporate coordinated care approaches to effectively assess and manage comorbidities in outpatient settings to decrease unnecessary hospitalizations. One such approach is Comprehensive Geriatric Assessment (CGA) which collects several patient-specific variables such as comorbidities, chronic diseases, functional status, polypharmacy, dietary status, psychosocial assessment, and family support^25^. Such assessment could help to evaluate the feasibility of managing cancer-related complications and comorbidities in oncology clinics^25^. In addition, prehabilitation techniques that have traditionally been employed for improving health status prior to surgeries could also be used for cancer patients for optimizing health status before cancer treatment, which could decrease avoidable hospitalization^26^. Reinforcing these patient-centered interventions, policy, and organizational level initiatives such as financial incentives for health care providers for decreasing avoidable hospitalization could help in significantly decreasing unwanted hospitalizations.

## Limitations

Although our study measured total cancer-related hospitalization, we were unable to identify preventable hospitalization. Such information could help future researchers to develop strategies for decreasing the health care burden associated with such hospitalizations. Though administrative databases are meticulously quality controlled, they are still susceptible to coding errors. CCS codes were used for identifying cancer hospitalization and there could be some coding errors leading to misclassification bias which could have affected our findings. NIS being an administrative database, it does not have information about cancer grading and staging. Therefore, it was not possible to ascertain active cancer versus symptom-free survival, which could have improved our findings. In addition, the NIS database deleted all personal identifiers to ensure the confidentiality of the data. Patients who were readmitted were considered as independent new admissions, thus obliterating the differences between index cases and readmitted cases. This could have caused an overestimation of hospitalization rates in our study. We used both primary and secondary diagnosis of cancer as inclusion criteria for the study. This could have led to some misclassification bias since some hospitalizations could be for conditions not related to cancer though there would have been a history of cancer. We included secondary diagnosis of cancer as well because, a history of cancer could affect the adverse outcome of hospitalizations.

## Conclusions

Findings of our study provide preliminary estimates and factors for developing an evidence base for decreasing cancer-related hospitalization. Our findings could also function as baseline estimates for comparing the effectiveness of initiatives to decrease cancer-related hospitalization in the future. Adopting integrated measures for managing cancer, treatment-related complications, and comorbidities through outpatient services could significantly aid in decreasing cancer-related hospitalization.

## Electronic supplementary material

Below is the link to the electronic supplementary material.

Supplementary Material 1

## Data Availability

Data is publicly available for purchase at: https://hcup-us.ahrq.gov/db/nation/nis/nisdbdocumentation.jsp. Please contact corresponding author at mrube001@fiu.edu for further details.
