# Supplementary material for: Trend and burden of adult cancer-related hospitalizations in the United States
Source: Sci Rep. 2025 Apr 18;15:13388. doi: 10.1038/s41598-025-97310-x (PMC12008368; doi:10.1038/s41598-025-97310-x)
Supplement: Supplementary file 1 — Supplementary Material 1 [file 41598_2025_97310_MOESM1_ESM.docx]

Supplementary Table 1. Top 10 primary reasons for cancer-related emergency department visits by cancer type

| **Cancer Type** | **Diagnoses** | **Weighted frequency (%)** |
| --- | --- | --- |
| Breast | Osteoarthritis | 331389 (5.0) |
|  | Congestive heart failure; nonhypertensive | 258846 (3.9) |
|  | Pneumonia (except that caused by tuberculosis or sexually transmitted disease) | 229062 (3.5) |
|  | Cardiac dysrhythmias | 226867 (3.5) |
|  | Septicemia (except in labor) | 216842 (3.3) |
|  | Complications of surgical procedures or medical care | 168919 (2.6) |
|  | Chronic obstructive pulmonary disease and bronchiectasis | 168805 (2.6) |
|  | Complication of device; implant or graft | 160386 (2.4) |
|  | Urinary tract infections | 155092 (2.4) |
|  | Acute cerebrovascular disease | 143069 (2.2) |
| Secondary malignancies | Septicemia (except in labor) | 419203 (6.7) |
|  | Other nervous system disorders | 361156 (5.8) |
|  | Pneumonia (except that caused by tuberculosis or sexually transmitted disease) | 305966 (4.9) |
|  | Fluid and electrolyte disorders | 277126 (4.4) |
|  | Acute and unspecified renal failure | 232612 (3.7) |
|  | Intestinal obstruction without hernia | 206780 (3.3) |
|  | Complications of surgical procedures or medical care | 177301 (2.8) |
|  | Respiratory failure; insufficiency; arrest (adult) | 176971 (2.8) |
|  | Pathological fracture | 150801 (2.4) |
|  | Urinary tract infections | 149079 (2.4) |
| Prostate (and other male genital cancers) | Osteoarthritis | 237932 (4.2) |
|  | Congestive heart failure; nonhypertensive | 221475 (3.9) |
|  | Septicemia (except in labor) | 217383 (3.8) |
|  | Cardiac dysrhythmias | 207723 (3.6) |
|  | Pneumonia (except that caused by tuberculosis or sexually transmitted disease) | 200554 (3.5) |
|  | Coronary atherosclerosis and other heart disease | 193072 (3.4) |
|  | Essential hypertension | 178429 (3.1) |
|  | Chronic obstructive pulmonary disease and bronchiectasis | 146908 (2.6) |
|  | Acute myocardial infarction | 146694 (2.6) |
|  | Urinary tract infections | 144716 (2.5) |
| Lung (and other respiratory cancers) | Pneumonia (except that caused by tuberculosis or sexually transmitted disease) | 658021 (12.8) |
|  | Chronic obstructive pulmonary disease and bronchiectasis | 479724 (9.3) |
|  | Respiratory failure; insufficiency; arrest (adult) | 298817 (5.8) |
|  | Septicemia (except in labor) | 253879 (4.9) |
|  | Pleurisy; pneumothorax; pulmonary collapse | 187326 (3.6) |
|  | Complications of surgical procedures or medical care | 178378 (3.5) |
|  | Fluid and electrolyte disorders | 164199 (3.2) |
|  | Cardiac dysrhythmias | 157198 (3.0) |
|  | Congestive heart failure; nonhypertensive | 143696 (2.8) |
|  | Pulmonary heart disease | 94446 (1.8) |
| Colon | Intestinal obstruction without hernia | 311951 (7.5) |
|  | Complications of surgical procedures or medical care | 171860 (4.1) |
|  | Septicemia (except in labor) | 158090 (3.8) |
|  | Congestive heart failure; nonhypertensive | 152641 (3.7) |
|  | Pneumonia (except that caused by tuberculosis or sexually transmitted disease) | 134181 (3.2) |
|  | Cardiac dysrhythmias | 120773 (2.9) |
|  | Acute and unspecified renal failure | 116935 (2.8) |
|  | Other gastrointestinal disorders | 116841 (2.8) |
|  | Fluid and electrolyte disorders | 114543 (2.7) |
|  | Gastrointestinal hemorrhage | 114349 (2.7) |
| Cancer of unknown or unspecified origin | Septicemia (except in labor) | 225149 (5.9) |
|  | Pneumonia (except that caused by tuberculosis or sexually transmitted disease) | 178524 (4.7) |
|  | Congestive heart failure; nonhypertensive | 148404 (3.9) |
|  | Deficiency and other anemia | 140377 (3.7) |
|  | Complications of surgical procedures or medical care | 95696 (2.5) |
|  | Acute and unspecified renal failure | 94476 (2.5) |
|  | Chronic obstructive pulmonary disease and bronchiectasis | 93528 (2.5) |
|  | Cardiac dysrhythmias | 87234 (2.3) |
|  | Complication of device; implant or graft | 85805 (2.3) |
|  | Acute cerebrovascular disease | 80967 (2.1) |
| Female reproductive | Intestinal obstruction without hernia | 171024 (5.4) |
|  | Complications of surgical procedures or medical care | 136858 (4.3) |
|  | Septicemia (except in labor) | 106601 (3.3) |
|  | Urinary tract infections | 100263 (3.1) |
|  | Osteoarthritis | 79004 (2.5) |
|  | Other nutritional; endocrine; and metabolic disorders | 74200 (2.3) |
|  | Acute and unspecified renal failure | 73043 (2.3) |
|  | Fluid and electrolyte disorders | 72209 (2.3) |
|  | Essential hypertension | 70525 (2.2) |
|  | Pneumonia (except that caused by tuberculosis or sexually transmitted disease) | 69330 (2.2) |
| Melanoma (and other skin cancers) | Osteoarthritis | 235421 (8.5) |
|  | Cardiac dysrhythmias | 113314 (4.1) |
|  | Septicemia (except in labor) | 104200 (3.7) |
|  | Congestive heart failure; nonhypertensive | 98580 (3.5) |
|  | Pneumonia (except that caused by tuberculosis or sexually transmitted disease) | 96639 (3.5) |
|  | Spondylosis; intervertebral disc disorders; other back problems | 88658 (3.2) |
|  | Coronary atherosclerosis and other heart disease | 80333 (2.9) |
|  | Acute cerebrovascular disease | 76001 (2.7) |
|  | Complication of device; implant or graft | 75007 (2.7) |
|  | Acute myocardial infarction | 68508 (2.5) |
| Non-Hodgkin lymphoma | Septicemia (except in labor) | 171966 (7.4) |
|  | Pneumonia (except that caused by tuberculosis or sexually transmitted disease) | 139317 (6.0) |
|  | Diseases of white blood cells | 93187 (4.0) |
|  | Congestive heart failure; nonhypertensive | 91310 (3.9) |
|  | Deficiency and other anemia | 88064 (3.8) |
|  | Acute and unspecified renal failure | 67825 (2.9) |
|  | Cardiac dysrhythmias | 61909 (2.7) |
|  | Fluid and electrolyte disorders | 61604 (2.6) |
|  | Complication of device; implant or graft | 60047 (2.6) |
|  | Complications of surgical procedures or medical care | 47430 (2.0) |
| Leukemia(s) | Septicemia (except in labor) | 186382 (8.9) |
|  | Pneumonia (except that caused by tuberculosis or sexually transmitted disease) | 171368 (8.2) |
|  | Deficiency and other anemia | 151344 (7.3) |
|  | Diseases of white blood cells | 99721 (4.8) |
|  | Congestive heart failure; nonhypertensive | 74799 (3.6) |
|  | Complication of device; implant or graft | 70764 (3.4) |
|  | Acute and unspecified renal failure | 59445 (2.8) |
|  | Coagulation and hemorrhagic disorders | 45175 (2.2) |
|  | Cardiac dysrhythmias | 44661 (2.1) |
|  | Fluid and electrolyte disorders | 41190 (2.0) |
| Bladder (and other urinary organ cancers) | Septicemia (except in labor) | 108400 (5.9) |
|  | Urinary tract infections | 105428 (5.7) |
|  | Acute and unspecified renal failure | 86644 (4.7) |
|  | Complications of surgical procedures or medical care | 77882 (4.2) |
|  | Congestive heart failure; nonhypertensive | 67924 (3.7) |
|  | Chronic obstructive pulmonary disease and bronchiectasis | 63180 (3.4) |
|  | Genitourinary symptoms and ill-defined conditions | 60258 (3.3) |
|  | Pneumonia (except that caused by tuberculosis or sexually transmitted disease) | 59438 (3.2) |
|  | Cardiac dysrhythmias | 53937 (2.9) |
|  | Other diseases of kidney and ureters | 53824 (2.9) |
| Gastrointestinal | Septicemia (except in labor) | 88647 (5.3) |
|  | Complications of surgical procedures or medical care | 79331 (4.8) |
|  | Fluid and electrolyte disorders | 74108 (4.4) |
|  | Gastrointestinal hemorrhage | 65504 (3.9) |
|  | Intestinal obstruction without hernia | 65176 (3.9) |
|  | Biliary tract disease | 63800 (3.8) |
|  | Pneumonia (except that caused by tuberculosis or sexually transmitted disease) | 60195 (3.6) |
|  | Nutritional deficiencies | 52175 (3.1) |
|  | Acute and unspecified renal failure | 44966 (2.7) |
|  | Deficiency and other anemia | 39115 (2.3) |
| Kidney and renal | Acute and unspecified renal failure | 67982 (4.4) |
|  | Complications of surgical procedures or medical care | 60133 (3.9) |
|  | Congestive heart failure; nonhypertensive | 55702 (3.6) |
|  | Essential hypertension | 54840 (3.6) |
|  | Septicemia (except in labor) | 50954 (3.3) |
|  | Pneumonia (except that caused by tuberculosis or sexually transmitted disease) | 47837 (3.1) |
|  | Cardiac dysrhythmias | 41015 (2.7) |
|  | Fluid and electrolyte disorders | 36622 (2.4) |
|  | Chronic obstructive pulmonary disease and bronchiectasis | 36505 (2.4) |
|  | Urinary tract infections | 33859 (2.2) |
| Head and neck | Complications of surgical procedures or medical care | 66580 (5.5) |
|  | Pneumonia (except that caused by tuberculosis or sexually transmitted disease) | 65879 (5.4) |
|  | Septicemia (except in labor) | 55853 (4.6) |
|  | Chronic obstructive pulmonary disease and bronchiectasis | 53324 (4.4) |
|  | Fluid and electrolyte disorders | 50095 (4.1) |
|  | Respiratory failure; insufficiency; arrest (adult) | 46923 (3.9) |
|  | Aspiration pneumonitis; food/vomitus | 44617 (3.7) |
|  | Other gastrointestinal disorders | 28750 (2.4) |
|  | Nutritional deficiencies | 28259 (2.3) |
|  | Essential hypertension | 27500 (2.3) |
| Rectal | Intestinal obstruction without hernia | 96022 (8.1) |
|  | Other gastrointestinal disorders | 94164 (7.9) |
|  | Complications of surgical procedures or medical care | 82309 (6.9) |
|  | Septicemia (except in labor) | 45652 (3.8) |
|  | Gastrointestinal hemorrhage | 40468 (3.4) |
|  | Fluid and electrolyte disorders | 39447 (3.3) |
|  | Complication of device; implant or graft | 37605 (3.2) |
|  | Acute and unspecified renal failure | 35802 (3.0) |
|  | Essential hypertension | 30170 (2.5) |
|  | Urinary tract infections | 24254 (2.0) |
| Multiple myeloma | Septicemia (except in labor) | 84945 (8.1) |
|  | Acute and unspecified renal failure | 82044 (7.8) |
|  | Pneumonia (except that caused by tuberculosis or sexually transmitted disease) | 78493 (7.5) |
|  | Deficiency and other anemia | 62193 (5.9) |
|  | Pathological fracture | 45790 (4.4) |
|  | Congestive heart failure; nonhypertensive | 41820 (4.0) |
|  | Fluid and electrolyte disorders | 31848 (3.0) |
|  | Complication of device; implant or graft | 25492 (2.4) |
|  | Cardiac dysrhythmias | 23507 (2.2) |
|  | Other nervous system disorders | 22415 (2.1) |
| Pancreatic | Biliary tract disease | 97741 (11.3) |
|  | Septicemia (except in labor) | 65099 (7.5) |
|  | Fluid and electrolyte disorders | 46352 (5.3) |
|  | Pancreatic disorders (not diabetes) | 29116 (3.4) |
|  | Acute and unspecified renal failure | 26663 (3.1) |
|  | Complications of surgical procedures or medical care | 26616 (3.1) |
|  | Other liver diseases | 26479 (3.1) |
|  | Complication of device; implant or graft | 25793 (3.0) |
|  | Nutritional deficiencies | 25155 (2.9) |
|  | Phlebitis; thrombophlebitis and thromboembolism | 23198 (2.7) |
| Brain and nervous system | Other nervous system disorders | 121581 (17.0) |
|  | Epilepsy; convulsions | 88562 (12.4) |
|  | Rehabilitation care; fitting of prostheses; and adjustment of devices | 32580 (4.5) |
|  | Acute cerebrovascular disease | 29012 (4.1) |
|  | Septicemia (except in labor) | 21420 (3.0) |
|  | Complications of surgical procedures or medical care | 19865 (2.8) |
|  | Fluid and electrolyte disorders | 18544 (2.6) |
|  | Essential hypertension | 17666 (2.5) |
|  | Paralysis | 16013 (2.2) |
|  | Pneumonia (except that caused by tuberculosis or sexually transmitted disease) | 14238 (2.0) |
| Thyroid | Thyroid disorders | 46028 (6.8) |
|  | Essential hypertension | 29175 (4.3) |
|  | Other nutritional; endocrine; and metabolic disorders | 26795 (4.0) |
|  | Osteoarthritis | 26603 (4.0) |
|  | Cardiac dysrhythmias | 18719 (2.8) |
|  | Complications of surgical procedures or medical care | 17357 (2.6) |
|  | Pneumonia (except that caused by tuberculosis or sexually transmitted disease) | 14682 (2.2) |
|  | Septicemia (except in labor) | 13208 (2.0) |
|  | Spondylosis; intervertebral disc disorders; other back problems | 12853 (1.9) |
|  | Congestive heart failure; nonhypertensive | 12048 (1.8) |
| Liver | Other liver diseases | 101525 (14.3) |
|  | Hepatitis | 49582 (7.0) |
|  | Septicemia (except in labor) | 48796 (6.9) |
|  | Biliary tract disease | 32734 (4.6) |
|  | Acute and unspecified renal failure | 29021 (4.1) |
|  | Fluid and electrolyte disorders | 22960 (3.2) |
|  | Gastrointestinal hemorrhage | 22200 (3.1) |
|  | Complication of device; implant or graft | 19060 (2.7) |
|  | Phlebitis; thrombophlebitis and thromboembolism | 17996 (2.5) |
|  | Complications of surgical procedures or medical care | 17310 (2.4) |
| Hodgkin's disease | Pneumonia (except that caused by tuberculosis or sexually transmitted disease) | 18958 (5.9) |
|  | Septicemia (except in labor) | 18520 (5.7) |
|  | Congestive heart failure; nonhypertensive | 13554 (4.2) |
|  | Deficiency and other anemia | 10972 (3.4) |
|  | Diseases of white blood cells | 10345 (3.2) |
|  | Complication of device; implant or graft | 10050 (3.1) |
|  | Coronary atherosclerosis and other heart disease | 8050 (2.5) |
|  | Cardiac dysrhythmias | 7179 (2.2) |
|  | Fluid and electrolyte disorders | 6645 (2.1) |
|  | Acute myocardial infarction | 6475 (2.0) |
| Cancer of bone and connective tissue | Complications of surgical procedures or medical care | 16571 (6.1) |
|  | Diseases of white blood cells | 12025 (4.4) |
|  | Septicemia (except in labor) | 11913 (4.4) |
|  | Essential hypertension | 11125 (4.1) |
|  | Other nervous system disorders | 9801 (3.6) |
|  | Deficiency and other anemia | 8380 (3.1) |
|  | Fluid and electrolyte disorders | 8199 (3.0) |
|  | Acute posthemorrhagic anemia | 6908 (2.5) |
|  | Complication of device; implant or graft | 6420 (2.4) |
|  | Respiratory failure; insufficiency; arrest (adult) | 6025 (2.2) |

Supplementary Table 2. Top primary reasons for cancer-related emergency department visits related to maintenance chemotherapy or radiotherapy.

| **Diagnoses** | **Weighted frequency (%)** |
| --- | --- |
| Deficiency and other anemia | 141047 (10.3) |
| Essential hypertension | 104939 (7.7) |
| Fluid and electrolyte disorders | 49225 (3.6) |
| Coagulation and hemorrhagic disorders | 46045 (3.4) |
| Diabetes mellitus without complication | 45079 (3.3) |
| Other nervous system disorders | 34182 (2.5) |
| Complications of surgical procedures or medical care | 33537 (2.5) |
| Other nutritional; endocrine; and metabolic disorders | 31197 (2.3) |
| Other gastrointestinal disorders | 30872 (2.3) |
| Phlebitis; thrombophlebitis and thromboembolism | 28610 (2.1) |

Supplementary Table 3. Trends of individual cancer types during the study period (rates per 100,000 cancer-related hospitalizations)

| **Cancer type** | **2008** | **2009** | **2010** | **2011** | **2012** | **2013** | **2014** | **2015** | **2016** | **2017** | **2018** | **2019** |
| --- | --- | --- | --- | --- | --- | --- | --- | --- | --- | --- | --- | --- |
| Head and neck | 2213 (2081-2344) | 2212 (2077-2347) | 2100 (1978-2221) | 2140 (2021-2259) | 2229 (2084-2375) | 2292 (2129-2454) | 2139 (2011-2268) | 2237 (2159-2315) | 2239 (2159-2319) | 2227 (2155-2300) | 2195 (2124-2265) | 2152 (2079-2224) |
| Gastrointestinal | 3057 (2955-3158) | 2925 (2849-3001) | 3015 (2908-3121) | 2984 (2886-3082) | 2964 (2874-3054) | 3039 (2928-3149) | 2977 (2875-3078) | 2990 (2934-3045) | 3013 (2959-3068) | 2970 (2915-3025) | 2913 (2861-2965) | 2962 (2910-3015) |
| Colon | 8614 (8313-8914) | 8537 (8259-8815) | 7935 (7464-8407) | 7761 (7394-8129) | 7722 (7447-7997) | 7246 (6899-7593) | 7481 (7175-7787) | 7333 (7176-7490) | 7202 (7049-7355) | 7016 (6873-7160) | 6751 (6614-6888) | 6565 (6440-6690) |
| Rectal | 2256 (2182-2330) | 2251 (2176-2326) | 2257 (2178-2335) | 2143 (2066-2220) | 2149 (2070-2228) | 2055 (1984-2125) | 2112 (2038-2187) | 2069 (2024-2114) | 2107 (2061-2154) | 2171 (2124-2217) | 2142 (2097-2187) | 2096 (2050-2143) |
| Liver | 925 (821-1029) | 911 (827-994) | 1025 (924-1125) | 1163 (1010-1317) | 1200 (1074-1327) | 1235 (1114-1356) | 1236 (1105-1367) | 1340 (1272-1407) | 1424 (1357-1491) | 1471 (1406-1536) | 1563 (1494-1632) | 1583 (1517-1650) |
| Pancreatic | 1477 (1413-1541) | 1502 (1442-1562) | 1513 (1450-1576) | 1586 (1508-1663) | 1514 (1442-1585) | 1596 (1505-1686) | 1561 (1472-1651) | 1537 (1494-1580) | 1556 (1509-1604) | 1584 (1539-1629) | 1573 (1530-1616) | 1612 (1567-1658) |
| Lung (and other respiratory cancers) | 10519 (10168-10870) | 10296 (9985-10606) | 9883 (9469-10296) | 9651 (9266-10036) | 9682 (9351-10013) | 9069 (8720-9419) | 9078 (8753-9404) | 9121 (8945-9298) | 8936 (8761-9110) | 8584 (8422-8746) | 8315 (8163-8467) | 8055 (7915-8195) |
| Cancer of bone and connective tissue | 477 (404-550) | 445 (389-501) | 514 (406-622) | 551 (458-644) | 473 (414-531) | 534 (440-628) | 485 (424-546) | 519 (475-562) | 510 (466-554) | 525 (483-568) | 511 (470-552) | 466 (428-503) |
| Melanoma (and other skin cancers) | 3188 (3020-3356) | 3385 (3195-3574) | 3479 (3246-3712) | 3893 (3669-4116) | 4539 (4199-4879) | 4825 (4402-5248) | 5030 (4741-5319) | 5240 (5071-5409) | 5596 (5416-5776) | 6073 (5885-6260) | 6564 (6367-6762) | 7000 (6793-7207) |
| Breast | 12040 (11732-12347) | 12030 (11701-12358) | 11643 (11161-12126) | 11798 (11321-12276) | 12186 (11788-12583) | 12024 (11570-12477) | 12350 (11929-12771) | 12250 (12023-12477) | 11952 (11725-12180) | 11586 (11366-11807) | 11468 (11258-11679) | 11253 (11053-11453) |
| Female reproductive | 6141 (5851-6431) | 5874 (5605-6143) | 5733 (5458-6007) | 5845 (5555-6136) | 5956 (5715-6198) | 5832 (5580-6084) | 5625 (5397-5852) | 5687 (5562-5812) | 5606 (5481-5731) | 5776 (5647-5905) | 5628 (5507-5749) | 5552 (5437-5666) |
| Prostate (and other male genital cancers) | 10224 (9916-10532) | 10675 (10365-10985) | 10396 (9920-10871) | 10621 (10203-11039) | 10807 (10442-11173) | 10343 (9907-10778) | 10966 (10603-11328) | 10384 (10186-10582) | 10141 (9947-10335) | 9868 (9685-10051) | 9812 (9634-9990) | 9813 (9640-9986) |
| Bladder (and other urinary organ cancers) | 3270 (3172-3368) | 3355 (3250-3460) | 3141 (3013-3268) | 3189 (3073-3306) | 3288 (3189-3388) | 3229 (3112-3346) | 3374 (3263-3484) | 3319 (3256-3381) | 3320 (3256-3384) | 3355 (3295-3416) | 3338 (3278-3399) | 3336 (3278-3394) |
| Kidney and renal | 2360 (2294-2425) | 2495 (2423-2567) | 2571 (2492-2650) | 2687 (2609-2765) | 2731 (2636-2826) | 2733 (2645-2821) | 2882 (2787-2977) | 2858 (2806-2910) | 2898 (2847-2950) | 2929 (2880-2978) | 2970 (2920-3019) | 2963 (2913-3013) |
| Brain and nervous system | 1332 (1220-1445) | 1344 (1204-1484) | 1324 (1201-1446) | 1311 (1182-1440) | 1217 (1120-1314) | 1325 (1219-1431) | 1289 (1131-1447) | 1269 (1205-1334) | 1268 (1203-1333) | 1287 (1221-1353) | 1293 (1228-1359) | 1325 (1258-1392) |
| Thyroid | 1233 (1154-1312) | 1247 (1162-1332) | 1268 (1175-1361) | 1337 (1241-1433) | 1211 (1139-1283) | 1314 (1217-1411) | 1365 (1268-1462) | 1335 (1291-1379) | 1298 (1256-1339) | 1287 (1251-1323) | 1240 (1207-1273) | 1247 (1215-1278) |
| Hodgkin's disease | 650 (617-683) | 634 (603-665) | 620 (585-656) | 599 (568-629) | 610 (575-646) | 568 (539-596) | 533 (506-561) | 572 (552-593) | 546 (528-565) | 559 (541-577) | 555 (536-573) | 543 (525-561) |
| Non-Hodgkin lymphoma | 4290 (4191-4390) | 4252 (4161-4342) | 4255 (4147-4364) | 4225 (4122-4328) | 4138 (4043-4234) | 4177 (4089-4265) | 4152 (4058-4246) | 4159 (4101-4216) | 4197 (4139-4255) | 4153 (4095-4210) | 4123 (4062-4183) | 4017 (3956-4077) |
| Leukemia(s) | 3556 (3407-3706) | 3556 (3432-3679) | 3624 (3332-3915) | 3704 (3424-3983) | 3639 (3436-3842) | 3553 (3387-3718) | 3592 (3425-3759) | 3716 (3583-3849) | 3850 (3721-3980) | 3864 (3735-3994) | 3902 (3769-4034) | 4045 (3930-4161) |
| Multiple myeloma | 1890 (1729-2052) | 1739 (1661-1817) | 1781 (1695-1867) | 1810 (1680-1939) | 1770 (1638-1903) | 1804 (1668-1941) | 1831 (1742-1920) | 1843 (1786-1900) | 1950 (1889-2012) | 1933 (1873-1993) | 1998 (1937-2060) | 2019 (1958-2079) |
| Cancer of unknown or unspecified origin | 5489 (5355-5624) | 5734 (5599-5868) | 6395 (6102-6689) | 6696 (6493-6899) | 6874 (6701-7046) | 6914 (6651-7176) | 7017 (6849-7184) | 7065 (6951-7178) | 7082 (6973-7190) | 7313 (7199-7427) | 7433 (7320-7546) | 7504 (7401-7607) |
| Secondary malignancies | 11829 (11546-12112) | 11516 (11213-11819) | 12204 (11158-13250) | 11148 (10648-11648) | 10355 (10049-10661) | 11325 (10232-12418) | 10670 (10232-11107) | 10588 (10298-10877) | 10813 (10517-11110) | 11106 (10791-11421) | 11455 (11151-11758) | 11717 (11423-12011) |
| Maintenance chemotherapy and radiotherapy | 2969 (2591-3346) | 3087 (2689-3484) | 3323 (2577-4069) | 3158 (2465-3850) | 2742 (2348-3136) | 2970 (2508-3433) | 2254 (1942-2566) | 2570 (2294-2845) | 2493 (2233-2753) | 2362 (2126-2598) | 2258 (2033-2483) | 2175 (1992-2358) |
